# High-level cytoplasmic claudin 3 expression is an independent predictor of poor survival in triple-negative breast cancer

**DOI:** 10.1186/s12885-018-4141-z

**Published:** 2018-02-27

**Authors:** Anniina Jääskeläinen, Ylermi Soini, Arja Jukkola-Vuorinen, Päivi Auvinen, Kirsi-Maria Haapasaari, Peeter Karihtala

**Affiliations:** 1 Department of Oncology and Radiotherapy, Medical Research Center Oulu, Oulu University Hospital and University of Oulu, P.O. Box 22, 90029 Oulu, Finland; 2 Department of Pathology, Medical Research Center, Oulu University Hospital, Oulu, Finland; 3 Department of Pathology, University of Eastern Finland, Kuopio, Finland; 4 Department of Oncology, and Cancer Center, Kuopio University Hospital, and Institute of Clinical Medicine, University of Eastern Finland, Kuopio, Finland

**Keywords:** Breast cancer, Claudin, Epithelial-to-mesenchymal transition, Immunohistochemistry, Prognosis

## Abstract

**Background:**

The subtype of claudin-low breast cancer can be reliably determined only by gene-expression profiling. Attempts have been made to develop immunohistochemical surrogates, which nearly always focus on membranous claudin expression.

**Methods:**

We assessed the immunohistochemical expression of both membranous and cytoplasmic claudins 3, 4 and 7 in a series of 197 non-metastatic breast cancers, enriched with triple-negative breast cancers (TNBCs; 60%). The expression of epithelial-to-mesenchymal transition-regulating transcription factors Sip1, Zeb1 and vimentin had previously been determined in the same material.

**Results:**

In multivariate analysis, strong cytoplasmic claudin 3 expression was associated with poor relapse-free survival (RFS), disease-free survival, distant disease-free survival, breast cancer-specific survival and overall survival among TNBC patients (for RFS, RR 5.202, 95% CI 1.210–22.369, *p* = 0.027, vs. T-class, RR 0.663, 95% CI 0.168–2.623, *p* = 0.558, and N-class, RR 3.940, 95% CI 0.933–16.631, *p* = 0.062). Cytoplasmic claudin 3 expression was also associated with strong nuclear Sip1 expression (*p* = 0.000053), TNBC phenotype (*p* = 0.012) and within them, non-basal-like phenotype (*p* = 0.026). Cytoplasmic claudin 7 was associated with dismal RFS (RR 6.328, 95% CI 1.401–28.593, *p* = 0.016, vs. T-class, RR 0.692, 95% CI 0.242–1.982, *p* = 0.493, and N-class, RR 2.981, 95% CI 1.1016–8.749, *p* = 0.047). Low cytoplasmic expression of claudins 3, 4 and 7 together also predicted poor RFS (RR 6.070, 95% CI 1.347–27.363, *p* = 0.019, vs. T-class, RR 0.677, 95% CI 0.237–1.934, *p* = 0.467, and N-class, RR 3.167, 95% CI 1.079–9.290, *p* = 0.036).

**Conclusions:**

Immunohistochemical expression levels of cytoplasmic claudins 3 and 7 appear to be novel prognostic factors in TNBC.

## Background

Claudins are tight junctional proteins of 20–24 kDa that are present on the apicolateral membranes of epithelial, endothelial and mesothelial cells [1, 2]. They have barrier and fence functions, and they take part in signal transduction [2]. They have four transmembranous domains and the molecules form two extracellular loops that harbor sites for functions of claudins such as paracellular permeability and attachment sites for *Clostridium perfringens* toxin or hepatitis C virus [1, 2]. The intracytoplasmic carboxyterminal part of the molecule has PDZ domains for attachment to ZO1–3, by which claudins may influence cellular signaling [3]. There are 27 different claudins known [4].

In malignancies, the expression of claudins varies, depending on the site and type of the tumor [5] and claudins can be used in the differential diagnosis of tumors in some cases. As an example, claudins 3 and 4 are very likely to be expressed in metastatic carcinomas of the pleura, while mesotheliomas are usually negative, and the expression of claudins 4 and 7 has been suggested to differentiate cholangiocarcinoma and hepatocellular carcinoma [6, 7]. Claudins 3 and 4 are particularly overexpressed in several carcinomas, including breast cancer [2].

In addition to barrier and fence functions, individual claudins harbor different properties affecting tumor growth and spread. Claudin 4 has been shown to induce angiogenesis, the spread and proliferation of MCF-7 cells, while abrogating apoptosis [8, 9]. Claudin 4 appears to be overexpressed frequently in metastatic breast cancer tissues compared with primary sites [10]. Inhibiting claudin 3 overexpression in MCF-7 cells has resulted in decreased tumor cell migration [11]. Claudins may also influence the prognosis of tumors. Low-level claudin 4 expression is associated with poor prognosis in esophageal and pancreatic carcinoma [12, 13]. On the other hand, claudin 3 overexpression is an indicator of poor prognosis in serous ovarian carcinoma, while its downregulation predicts poor survival in squamous cell lung carcinoma [14, 15]. Low-level claudin 7 expression is associated with better prognosis of patients with oral squamous cell carcinoma [16], and in prostate carcinoma, with increased tumor grade [17].

Triple-negative breast cancer (TNBC) is a subtype with lack of expression of ER, PR and HER2 and it constitutes of about 15–20% of cancer cases [18]. TNBCs are enriched in basal-like (BLBC) and claudin-low breast cancer molecular subtypes, the former expressing basal cell markers and the latter, in addition to low claudin 3, 4, 7 and E-cadherin expression, showing induced expression of EMT (epithelial-to-mesenchymal transition)-related genes, immune system-related genes and stem-cell features [18, 19]. The estimated incidence of claudin-low breast cancer is 7–14% and long-term prognosis is relatively poor [19–21].

The clinical research on claudins in cancers is rapidly growing and monoclonal claudin antibodies have also shown promising results in a phase II trial in cases of gastric cancer [22]. The clinical benefit of finding this subgroup in breast cancer is still limited, since identifying a tumor as a claudin-low subtype requires gene expression profiling from fresh frozen tumor material. Different approaches to define claudin-low subtypes by immunohistochemistry (IHC) have been proposed, but none have been validated in independent cohorts.

Claudins thus have various biological and pathological properties, depending on their specific subtypes and localization. Previous claudin protein expression studies in breast cancer have mainly been concentrated on membranous claudin expression and/or have not involved the expression of separate claudins. We aimed to clarify if the expression of claudins 3, 4 and 7, in membranes and cytoplasm, could be associated with the outcome of the disease. Since claudins are overexpressed in TNBCs, we used TNBC-enriched material, previously assessed for expression of major EMT regulators.

## Methods

There was a total of 197 women with non-metastatic breast cancer in the research material (Table 1). Of these, 119 were TNBC cases (60.4%) and 78 non-TNBC. Of 99 evaluable TNBC cases, 87 (73.1%) showed a basal-like phenotype as they expressed either CK5/6 or EGFR-1. The median follow-up time was 100.0 months (mean 94.0 months).

**Table 1:** Patient material

	N (%)
Breast cancer type	197 (100.0%)
TNBC	119 (60.4%)
Non-TNBC	78 (39.6%)
Histopathology	197 (100.0%)
Ductal	176 (89.3%)
Lobular	4 (2.0%)
Medullary	10 (5.1%)
Tubular	2 (1.0%)
Other	5 (2.5%)
Histopathological grade	197 (100.0%)
Grade 1	5 (2.5%)
Grade 2	42 (21.3%)
Grade 3	150 (76.1%)
ER status	197 (100.0%)
Negative (0%)	119 (60.4%)
Weak (1–9%)	0 (0.0%)
Moderate (10–59%)	14 (7.1%)
High (> 59%)	64 (32.5%)
PR status	197 (100.0%)
Negative (0%)	119 (60.4%)
Weak (1–9%)	0 (0.0%)
Moderate (10–59%)	31 (15.7%)
High (> 59%)	47 (23.9%)
Ki67 status	197 (100.0%)
Negative (< 5%)	11 (5.6%)
Weak (5–14%)	29 (14.7%)
Moderate (15–30%)	37 (18.8%)
High (> 30%)	64 (32.5%)
Missing	56 (28.4%)
T class	197 (100.0%)
T1	88 (44.7%)
T2	97 (49.2%)
T3	9 (4.6%)
T4	3 (1.5%)
N class	197 (100.0%)
N0	108 (54.8%)
N1	66 (33.5%)
N2	17 (8.6%)
N3	6 (3.0%)
M class	197 (100.0%)
M0	197 (100.0%)
Local relapse	197 (100.0%)
No local relapse	182 (92.4%)
Local relapse	15 (7.6%)
Distant metastases	197 (100.0%)
No distant metastases	145 (73.6%)
Distant metastases	52 (26.4%)

The specimen fixation, storing and staging was performed as previously described [23]. Tumor differentiation was classified according to the WHO Classification of Tumors [24].

### Immunohistochemistry

Claudin primary antibodies, designed for formalin-fixed paraffin-embedded tissue sections, were purchased from Zymed Laboratories Inc. (San Francisco, CA, USA). The antibodies used were polyclonal rabbit anti-claudin 3 (Z23.JM), monoclonal mouse anti-claudin 4 (clone 3E2C1), and polyclonal rabbit anti-claudin 7 (ZMD.241). Sections of 5 μm were deparaffinized and rehydrated. They were first heated in a microwave oven in tris-EDTA for 10 min and then incubated with the primary antibody for 60 min. The dilution was 1:50 for all anti-claudins and DAKO EnVision kits were used according to the manufacturer’s instructions for the detection of primary antibody. Color was developed by using diaminobenzidine, the sections were counterstained with hematoxylin and mounted with Pertex (Leica Microsystems, Germany). Negative controls were handled as previously described but with the primary antibody replaced by serum or PBS. Positive controls included tumor samples previously known to be positive for the claudins.

### Immunohistochemical scoring

Tumors exhibiting nuclear estrogen/progesterone receptor (ER or PR) expression in more than 9% of invasive tumor cells were considered as steroid receptor-positive. The TNBC group did not show any ER or PR positivity. In other words, tumors expressing ER or PR in 1–9% of invasive cells were excluded from the study. If a specimen exhibited a membranous HER2-positive result (1+ to 3+ on a scale of 0 to 3+) in IHC, HER2 gene amplification status was determined by means of chromogenic in situ hybridization. Breast cancers with six or more gene copies of HER2 in cells were considered HER2-positive. Expression of Ki-67 was studied by means of IHC as described previously [25]. The methods and results concerning cytokeratin 5/6, epidermal growth factor receptor and EMT marker immunostaining and assessment in this material have also been reported earlier [23]. The triple-negative tumors that also expressed either EGFR and/or CK5/6 were classified as basal-like breast cancers [25–27].

Claudin immunoreactivity was assessed semiquantitatively by dividing the immunoreactivity into five groups: 0–5%, 5–25%, 25–50%, 50–75% and over 75% positive. Membranous and cytoplasmic expression were assessed separately. For claudins 3 and 4 less than 50% positivity was considered to be low expression. Since claudin 7 expression was significantly weaker, less than 5% was considered as low expression. Claudin-low breast cancers were defined as those having low membranous expression of claudins 3, 4 and 7. Claudin assessments were performed by an experienced histopathologist (YS), who was blind to the clinical data at the time of the analysis.

### Statistical analysis

Statistical analysis was performed using IBM SPSS Statistics software, v. 23.0.0.0 (IBM Corporation, Armonk, NY, USA). T-class was divided in statistical analyses to either T1 or T2–4, and nodal status to either positive or negative. Expression of Ki-67 was divided into 0–14% or > 14% and grade was either grade I–II or grade III in the analyses. The significance of associations was defined by using two-sided Pearson’s Chi-square tests. Kaplan–Meier curves with the log-rank test were applied in survival analysis. Disease-free survival (DFS), relapse-free (RFS), distant disease-free (DDFS), breast cancer-specific (BCSS) and overall (OS) survival were calculated from the time of diagnosis to disease recurrence at any site (DFS), in the ipsilateral breast, scar, or axilla (RFS), at distant sites (DDFS), to the time of confirmed breast cancer-related death (BCSS) or time of death from any cause (OS). Cox regression analysis was applied in multivariate analysis, where the most important traditional prognostic factors, T-class (T1 or T2–4) and N-class (N0 or N1–3), were included to the model. In all statistical analyses, *p*-values less than 0.05 were considered significant.

## Results

### Expression patterns

**Fig. 1:**
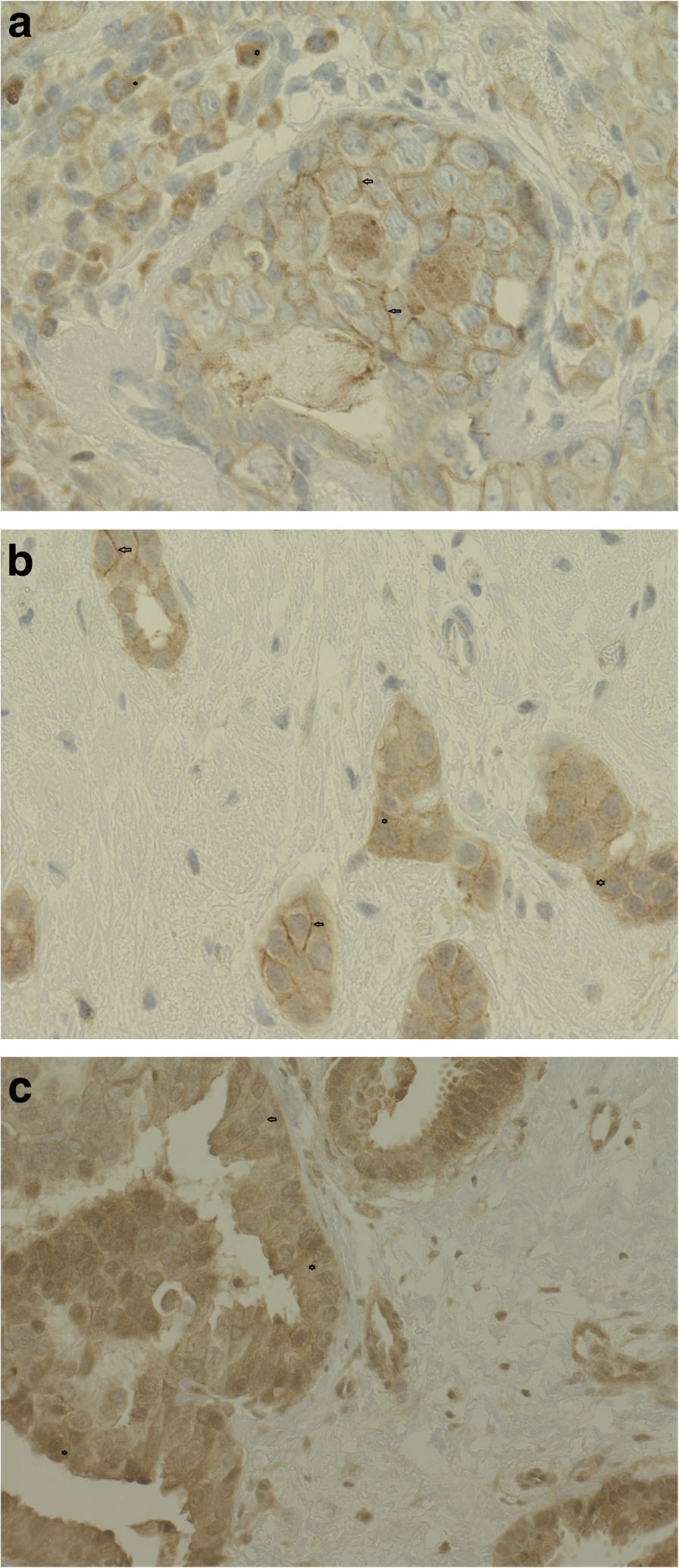
Immunohistochemical expression of claudins 3 (**a**), 4 (**b**) and 7 (**c**) in breast cancer. Asterisks demonstrate cytoplasmic and arrows membranous immunostaining

Among the total of 197 patients, claudin 3 was reliably assessable in 187 (94.9%) cases, claudin 4 in 191 (97.0%) and claudin 7 in 185 (93.9%) cases. Claudin expression is presented in Table 2 and examples of staining patterns are shown in Fig. 1.

**Table 2:** Expression levels of claudins 3, 4 and 7 in the whole material, separately in TNBC and non-TNBC groups and significance in comparison of the two groups (p-value, 2-sided Pearson’s chi-square test)

	Total N (%)	TNBC N (%)	Non-TNBC N (%)	*p*-value between TNBC and non-TNBC
Cytoplasmic Claudin 7	185 (100.0)	115 (100.0)	70 (100.0)	
CL 7 cytoplasmic 0–5%	98 (53.0)	51 (44.3)	47 (67.1)	0.0026
CL 7 cytoplasmic 6–100%	87 (47.0)	64 (55.7)	23 (32.9)	
Membranous Claudin 7	185 (100.0)	115 (100.0)	70 (100.0)	
CL7 membranous 0–5%	141 (76.2)	98 (85.2)	43 (61.4)	0.00023
CL 7 membranous 6–100%	44 (23.8)	17 (14.8)	27 (38.6)	
Cytoplasmic Claudin 4	191 (100.0)	116 (100.0)	75 (100.0)	
CL 4 cytoplasmic 0–50%	180 (94.2)	109 (94.0)	71 (94.7)	NS
CL 4 cytoplasmic 51–100%	11 (5.8)	7 (6.0)	4 (5.3)	
Membranous Claudin 4	191 (100.0)	116 (100.0)	75 (100.0)	
CL 4 membranous 0–50%	73 (38.2)	45 (38.8)	28 (37.3)	NS
CL 4 membranous 51–100%	118 (61.8)	71 (61.2)	47 (62.7)	
Cytoplasmic Claudin 3	187 (100.0)	111 (100.0)	76 (100.0)	
CL 3 cytoplasmic 0–50%	174 (93.0)	99 (89.2)	77 (98.7)	0.012
CL 3 cytoplasmic 51–100%	13 (7.0)	12 (10.8)	1 (1.3)	
Membranous Claudin 3	187 (100.0)	111 (100.0)	76 (100.0)	
CL 3 membranous 0–50%	99 (52.9)	63 (56.8)	36 (47.4)	NS
CL 3 membranous 51–100%	88 (47.1)	48 (43.2)	40 (52.6)	
Membranous claudin	190 (100.0)	114 (100.0)	76 (100.0)	
Low membranous expression of claudins 3, 4 and 7	37 (19.5)	25 (21.9)	12 (15.8)	NS
High membranous expression of at least one claudin	153 (80.5)	89 (78.1)	64 (84.2)	
Cytoplasmic claudin	174 (100.0)	106 (100.0)	68 (100.0)	
Low cytoplasmic expression of claudins 3, 4 and 7	91 (52.3)	46 (43.4)	45 (66.2)	0.0033
High cytoplasmic expression of at least one claudin	83 (47.7)	60 (56.6)	23 (33.8)

### Association with clinicopathological parameters

Cytoplasmic claudin 3 was overexpressed in TNBC tumors (*p* = 0.012), and within them, in non-basal-like TNBCs (*p* = 0.026) (Table 3). Likewise, cytoplasmic claudin 4 was associated with the non-basal-like phenotype of TNBCs (*p* = 0.00090). Stronger membranous claudin 3 expression was associated with patients with only bone metastases as the first metastatic site (*p* = 0.032).

**Table 3:** Significant 2-sided p-values of associations between claudin (cl) 3, 4 and 7 expression and traditional prognostic factors, EMT-regulating transcription factors and survival in univariate analysis

	Cyt cl 3	Membr cl 3	Cyt cl 4	Membr cl 4	Cyt cl 7	Membr cl 7	Membr claudin low	Cyt claudin low
T (T1 vs. T2–4)					0.0053			0.0074
N (N0 vs. N1–3)							0.013	
Grade (I–II vs. III)					0.043			0.013
TNBC	0.012				0.0026	0.00023		0.0033
BLBC	0.026		0.00090		0.023			0.0050
Ki67 (0–14% vs. > 14%)						0.0042		
1st metastatic site only in bone		0.032			0.016			
Zeb1						0.010		
Vimentin						0.042		
Cytoplasmic Sip1					0.0012			0.0029
Nuclear Sip1	0.000053							0.0029
DFS	0.0009							
DDFS	0.006							
RFS	0.000011							0.0033
BCSS	0.001							
OS	0.018							

The directions of the associations are described in the Results section. Cyt, cytoplasmic; Membr, membranous; TNBC, triple-negative breast cancer; BLBC, basal-like breast cancer; DFS, disease-free survival; DDFS, distant disease-free survival; RFS, relapse-free survival; BCSS, breast cancer-specific survival; OS, overall survival

Cytoplasmic claudin 7 expression was associated with smaller tumor size (*p* = 0.0053), better differentiation (*p* = 0.043) and with sites other than bone as the first metastatic site (*p* = 0.016). Cytoplasmic claudin 7 was also overexpressed in TNBC tumors (*p* = 0.0026), and within them, in non-basal-like TNBCs (*p* = 0.023). Membranous claudin 7 expression showed an inverse association with proliferation rate (*p* = 0.0042) and it was overexpressed in TNBC tumors (*p* = 0.00023).

Low-level membranous expression of claudins 3, 4 and 7 together was associated with node negativity (*p* = 0.013). Low-level expression of claudins 3, 4 and 7 together in cytoplasm was connected with higher grade (p = 0.013), larger primary tumor (*p* = 0.0074), a non-TNBC phenotype (*p* = 0.0033), and within TNBCs it was strongly connected with basal-like breast cancers (*p* = 0.0050).

### Associations between claudins and EMT-regulating transcription factors

Zeb1 expression in cancer cells was associated inversely with membranous claudin 7 expression (*p* = 0.010), while relatively strong cytoplasmic claudin 7 expression was associated with increased cytoplasmic Sip1 expression (*p* = 0.0012). An association between cytoplasmic claudin 3 and nuclear Sip1 expression was extremely significant (*p* = 0.0000053). Low-level expression of claudins 3, 4 and 7 in cytoplasm was associated with low levels of cytoplasmic and nuclear Sip1 expression (*p* = 0.0029 for both).

### Survival analysis

**Fig. 2:**
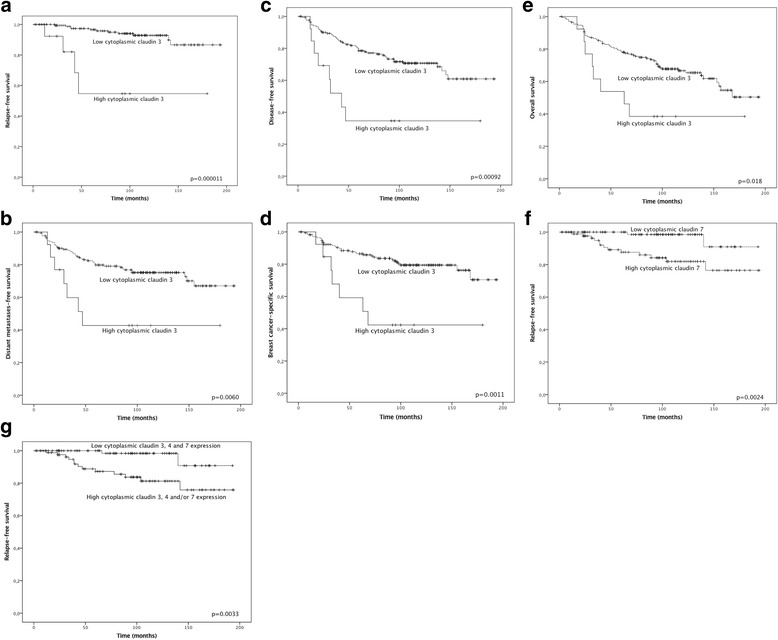
Kaplan–Meier curves of studied outcomes according to the expression of cytoplasmic claudin 3 (**a**–**e**), cytoplasmic claudin 7 (**f**) and claudins 3, 4 and 7 together (**g**). Crosses indicate censored cases

Cytoplasmic claudin 3 was associated with poor DFS (*p* = 0.0009), DDFS (*p* = 0.006), RFS (*p* = 0.00001), BCSS (*p* = 0.001) and OS (*p* = 0.018) in univariate analysis in the whole material (Fig. 2). However, since cytoplasmic claudin 3 was associated strongly with TNBC, and there was only one patient in the non-TNBC group with strong claudin 3 expression, the association between survival and cytoplasmic claudin 3 expression was significant only within TNBC patients (DFS, *p* = 0.0012; DDFS, *p* = 0.007; RFS, *p* = 0.000028; BCSS, *p* = 0.005; OS, *p* = 0.016).

In multivariate analysis, when T-class (T1 or T2–4) and N-class (N0 or N1–3) were taken into account, claudin 3 still remained a significant prognostic factor (Table 4). Notably, in RFS analysis the prognostic role of cytoplasmic claudin 3 (RR 6.162, 95% CI 1.785–21.272, *p* = 0.004) exceeded that of T-class (RR 0.714, 95% CI 0.240–2.124, *p* = 0.544) and N-class (RR 3.076, 95% CI 1.032–9.170, *p* = 0.044). When the analysis was performed separately in TNBC and non-TNBC cases, in TNBC strong cytoplasmic claudin 3 expression was solely an independent predictor of worse RFS (RR 5.202, 95% CI 1.210–22.369, *p* = 0.027, vs. T-class, RR 0.663, 95% CI 0.168–2.623, *p* = 0.558, and N-class RR 3.940, 95% CI 0.933–16.631, *p* = 0.062), while in cases of non-TNBC none of these three variables was an independent predictor of RFS in this model.

**Table 4:** Risk ratios, 95% confidence intervals and corresponding p-values concerning tumor size (T), nodal status (N) and cytoplasmic claudin 3 expression in multivariate analysis of disease-free survival (DFS), distant disease-free survival (DDFS), relapse-free survival (RFS), breast cancer-specific survival (BCSS) and overall survival (OS)

	T (T1 vs. T2–4)	N (N0 vs. N1–3)	Cytoplasmic claudin 3 expression (≤50% vs. > 50%)
DFS	2.721 (1.482–4.996); *p* = 0.0012	3.692 (2.072–6.590); *p* = 0.00001	3.897 (1.739–8.729); *p* = 0.00095
DDFS	4.245 (2.057–8.760); *p* = 0.000092	3.569 (1.894–6.724); *p* = 0.00083	3.655 (1.547–8.636); *p* = 0.0031
RFS	0.714 (0.240–2.124); *p* = 0.544	3.076 (1.032–9.170); *p* = 0.044	6.162 (1.785–21.272); *p* = 0.004
BCSS	3.596 (1.668–7.755); *p* = 0.0011	3.532 (1.731–7.204); *p* = 0.00052	3.881 (1.621–9.292); *p* = 0.0023
OS	2.118 (1.245–3.602); *p* = 0.0056	2.518 (1.522–4.165); *p* = 0.00032	2.394 (1.100–5.211); *p* = 0.028

Strong cytoplasmic claudin 7 expression was associated with poor RFS (*p* = 0.0024), and it was also significant in multivariate analysis (RR 6.328, 95% CI 1.401–28.593, *p* = 0.016, vs. T-class, RR 0.692, 95% CI 0.242–1.982, *p* = 0.493, and N-class, RR 2.981, 95% CI 1.106–8.749, *p* = 0.047). Stronger cytoplasmic claudin 4 expression predicted worse DDFS in non-TNBC patients (*p* = 0.017), but this did not remain significant in Cox regression analysis.

Low-level expression of claudins 3, 4 and 7 together in cytoplasm predicted poor RFS (*p* = 0.0033), being similar in TNBCs (*p* = 0.036) and non-TNBCs (*p* = 0.038). In Cox regression analysis, this remained as a significant factor (RR 6.070, 95% CI 1.347–27.363, *p* = 0.019, vs. T-class, RR 0.677, 95% CI 0.237–1.934, *p* = 0.467, and N-class, RR 3.167, 95% CI 1.079–9.290, p = 0.036).

## Discussion

Our aim was to establish if separate IHC assessment of the expression levels of claudins 3, 4 and 7 could be associated with different outcomes in breast cancer. Previous IHC studies concerning claudins in breast cancer have rarely involved both cytoplasmic and membranous claudin expression, but we decided to evaluate them separately. In addition, we had previously characterized major EMT-regulating transcription factors in most samples in the current material [28], which allowed us to correlate Sip1, Zeb1 and vimentin expression to the expression levels of claudins 3, 4 and 7. Other strengths of the current study were sufficient follow-up and the use of appropriate definition of TNBC, i.e. ER and PR cut-off levels were set at < 1% of nuclear expression.

Claudin-low breast cancers were first reported as a recognized subtype 10 years ago [29] but its IHC definition is still unclear. In a recent paper [21] the claudin-low subtype, defined as TNBC with low-level IHC expression two of four proteins (i.e. E-cadherin, and claudins 3, 4 and 7), was associated with exceedingly good RFS, with a local recurrence rate of only 1.3% at 10-year follow-up. Although not clear from the paper, it seems that only cytoplasmic claudin expression was examined. No associations between the expression levels of separate claudins to outcome were reported and the patient material was limited to T1 N0–T2 N0 patients. Lu et al. [30] defined claudin-low breast cancers as those with low claudin 1, 3, 4, 7 and 8 expression in IHC. Although low-level expression of all claudins was associated with disease recurrence, the subtype was not a significant predictor in multivariate analysis. In another IHC approach, claudin-low breast cancers were defined as those showing low-level claudin 3, 4, 7 and/or E-cadherin expression [31].

Cytoplasmic, dislocalized claudin 3 was of remarkable prognostic value in our material. Stronger cytoplasmic claudin 3 expression was also associated with TNBC subtype (compared with ER+/PR+/HER2- tumors), and within TNBCs, associated with the basal-like phenotype. Most notably, the prognostic value of cytoplasmic claudin 3 expression greatly exceeded that of T-class and N-class as a predictor of RFS, which was most remarkably shown among TNBC patients. Up to 33% of the women showing strong cytoplasmic claudin 3 expression, treated with modern surgical and oncological techniques, suffered a local recurrence during follow-up. In vitro data supports these results, since siRNA treatment against claudin 3 in metastatic breast cancer cells has been reported to attenuate cellular motility, and higher intracellular claudin 3 expression was connected to tight-junction disruption and local invasiveness. In colorectal cancer cells, claudin 3 overexpression promoted the malignant potential of cells, probably via epidermal growth factor-activated ERK1/2 and PI3K-Akt pathways [32]. Taken together, cytoplasmic localization of claudin 3 expression, in particular, appears to be a potential marker for predicting local recurrence. Membranous claudin 3 expression did not show prognostic significance, but it was associated with a tendency for the disease to have bone as a common first metastatic site.

In addition to having an essential role in tight-junction regulation, claudins regulate and are regulated by various oncogenes and tumor-promoting growth factors [2]. In resected squamocellular lung cancers, reduced claudin 3 expression has been found to be associated with increased vimentin protein expression and also poor survival [15]. As far as we know, no previous data exists on claudin in relation to vimentin, Zeb1 and Sip1 expression in breast cancers. High-level expression of Sip1 was earlier confirmed as a prognostic factor in terms of poor DFS in the current cohort [28], and now we report an extremely tight connection between cytoplasmic claudin 3 and nuclear Sip1 expression. Owing to this connection, low-level cytoplasmic expression claudins 3, 4 and 7 together was linked to nuclear Sip1 expression. It is possible that claudin 3 and Sip1 are partly regulated via the same mechanisms, although this has not yet been assessed. Since cytoplasmic but not membranous claudin 3 had a remarkable prognostic role, it could be hypothesized that this is due to EMT-mediated claudin regulation, cytoplasmic claudin expression reflecting out of place, aberrant claudin expression.

Low-level expression of claudin 7 was a significant predictor of better RFS in multivariate analysis, but, interestingly, it was also associated with TNBC subtype, larger primary tumor size and poorer differentiation. As in the context of claudin 3, correlation with survival was noted only when cytoplasmic expression was evaluated, while membranous expression did not have any prognostic significance, despite a strong inverse correlation between membranous claudin 7 expression, and proliferation. A putative correlation (*p* = 0.05 in univariate analysis) has been previously reported between membranous claudin 7 and RFS in a cohort of 75 breast carcinomas [33]. In another paper, membranous claudin 7 was also linked to poor survival, where the endpoints included both local and distant relapses [31]. An inverse correlation between claudin 7 expression and grade has also been reported in earlier studies [34, 35]. In colorectal cancer, increased claudin 7 expression has been associated with disruption of cell polarity, proliferation and tumor growth, both in vivo and in vitro, being in line with our breast cancer results [2]. Cytoplasmic Sip1 expression correlated tightly with that of cytoplasmic claudin 7. This interaction has not previously been assessed, but since previous evidence from colorectal cancer models suggests that claudin 7 promotes EMT, Sip1 may here be a connecting factor [36].

In the current material, high-level expression of cytoplasmic claudin 4 was associated with dismal DDFS in non-TNBC patients, but otherwise claudin 4 did not show associations with survival or clinicopathological parameters. Among TNBCs, claudin 4 was underexpressed in the BLBC phenotype as expected and has been previously reported [37]. This is discordant with some lines of previous evidence, where claudin 4 has been characterized as a risk factor of worse prognosis [38–41].

In the current study, we used the definition of claudin-low when expression of claudins 3 and 4 was recorded in less than 50% of malignant cells, and in the case of claudin 7, in less than 5% of cells. The cut-offs were roughly based on the median levels of expression of individual claudins. With this approach, 19.5% of cases met the criteria of membranous claudin-low breast cancer, but it should be underlined that in the current material we aimed to focus on claudin expression in TNBCs, which were therefore overrepresented and thus proportion cannot be directly compared with that in unselected materials. A membranous claudin-low phenotype in our material was associated with slightly less nodal involvement, but no other associations with clinicopathological parameters were observed. In a large DNA microarray study carried out by Sabatier et al. [20], no association between a claudin-low subtype and lymph node status was detected. Ma et al. previously reported membranous claudin 1 expression as a prognostic factor in terms of poor RFS and OS in a retrospective cohort consisting of TNBCs. In addition, they studied the expression of claudins 4 and 7, but no associations with survival were found [42]. In our patients low-level cytoplasmic expression of claudins 3, 4 and 7 together predicted worse RFS, a non-TNBC phenotype, larger primary tumors and poor differentiation. This “combined” parameter nevertheless did not have added prognostic or other value compared with the expression levels of individual claudins, especially cytoplasmic claudin 3.

## Conclusions

Immunohistochemical assessment of claudins offers a potentially more financially beneficial and convenient approach to distinguish the claudin-low subtype in breast cancer, when compared with gene expression profiling. On the basis of the current data, it seems that IHC expression of claudins 3 and 7, specifically in cytoplasm, could be used as novel prognostic factors in TNBCs. Although more studies are required to clarify their connections with EMT-regulating transcription factors, Sip1 and claudin regulation in particular seem to be interconnected in this context. Our results now need validation in a larger independent TNBC cohort. If confirmed, a clinical, randomized trial could be carried out to see if cytoplasmic claudin expression could be used in the adjuvant treatment selection process.

## Abbreviations

BCSS: Breast cancer-specific survival
DDFS: Distant disease-free survival
DFS: Disease-free survival
EMT: Epithelial to mesenchymal transition
ER: Estrogen receptor
HCC: Hepatocellular carcinoma
OS: Overall survival
PR: Progesterone receptor
RFS: Relapse-free survival
TNBC: Triple-negative breast cancer
